# Progesterone levels on hCG day and oocyte maturation in a Mexican IVF program

**DOI:** 10.5935/1518-0557.20240001

**Published:** 2024

**Authors:** Alfredo Cortés-Vazquez, Denisse Veliz-Figueroa, Karla Vargas-Estrada, Jesús-Daniel Moreno-García, Alfredo Cortés-Algara

**Affiliations:** 1 Reproductive Endocrinology Department, Centro Médico Nacional 20 de Noviembre, Mexico City, Mexico

**Keywords:** progesterone, oocyte, competence, maturation

## Abstract

**Objective:**

Does progesterone levels on hCG day influence maturation rates and number of mature oocytes during ovarian stimulation for IVF/ICSI cycles?.

**Methods:**

A retrospective, observational, analytic, cross-sectional and cohort study was performed at the Reproductive Endocrinology Department of the Centro Médico Nacional 20 de Noviembre in Mexico City between 2015 to 2020. All female patients underwent an ovarian stimulation cycle for IVF/ICSI, either with a mild or conventional stimulation protocol. Patients were classified according to their progesterone levels, Group 1 <1.5ng/ml and Group 2 >1.5mg/ml. A Spearman Rho test, a simple linear regression model, a Principal Component Analysis and a Student’s T-test, were performed.

**Results:**

A total of 600 patients were included. The overall results showed that there is a positive correlation between the number of retrieved, mature oocytes and progesterone levels on HCG day. After the Principal Component Analysis we observed that poor ovarian responders had the lowest maturation rate and number of mature oocytes. While the Student’s t test showed that progesterone levels beyond 1.5ng/ml are associated to a higher number of mature oocytes but not a better maturation rate.

**Conclusions:**

Higher serum progesterone levels are associated with increased retrieved and mature oocytes in high responders. At the same time, higher progesterone levels in lower responders are not associated with optimal ovarian response.

## INTRODUCTION

Over five million babies have been born worldwide using in vitro fertilization (IVF) techniques (van Loendersloot *et al*., 2014). This worldwide trend is not caused by a recent infertility pandemic but by increased access to IVF treatments. Contrary to the common belief, IVF does not guarantee success; around 38 to 49% of couples who start IVF cycles remain unfruitful, even if they undergo six IVF cycles (Malizia *et al*., 2009). Clinical studies have confirmed that the slight progesterone rise on HCG day negatively affects the pregnancy rate in a fresh cycle and will be reduced when progesterone increases to more than 1.5ng/ml. Interestingly, premature progesterone rise is more prevalent in non-white racial groups. The Latino population had an adjusted prevalence of 21.2% and an increased 2.16 odds ratio of premature progesterone rise (Hill *et al*., 2017).

Assisted conception protocols aim to induce multiple follicles that ultimately produce several oocytes and embryos to transfer. Several authors concluded in a faultless study that live-birth rates increase and remain relatively unchanged with an ovarian response between seven to twenty oocytes (Polyzos *et al*., 2018). There are circumstances in which oocyte maturation is indispensable since maturity influences fertilization rates (Jeve *et al*., 2018). In intracytoplasmic sperm injection (ICSI) cycles, only metaphase II (MII) oocytes are injected. Moreover, the goal of oocyte cryopreservation is generally to freeze mature oocytes for later thawing and fertilization with IVF (Lee *et al*., 2013). Current evidence shows that around 8.6% to 20% of oocytes retrieved after controlled ovarian stimulation are immature either at the germinal vesicle (GV) or metaphase I (MI) (Beall *et al*., 2010; Parrella *et al*., 2019).

According to the literature, Metaphase I oocytes (MI) have significantly reduced fertilization rates compared to MII oocytes (Jeve *et al*., 2018). Also, an increased proportion of immature oocytes (GV and MI) diminish the ability of the mature sibling oocytes to be regularly fertilized, arising in a diminished number of good-quality embryos with a lower ability to implant (Parrella *et al*., 2019). This reproductive event can impair pregnancy rates significantly in patients with low ovarian reserve.

Previous experience shows a negative influence of progesterone in oocyte maturation (Cortés-Vazquez *et al*., 2021; Salehnia & Zavareh, 2013). So it is reasonable to assess the impact of progesterone levels on HCG day on the oocyte maturation process.

This study aims to evaluate the correlations between progesterone levels on HCG day with the percentage of mature oocytes and the number of mature oocytes during ovarian stimulation for IVF/ICSI cycles.

## MATERIALS AND METHODS

### Study population and design

This retrospective, observational, analytic, cross-sectional and cohort study was performed at the Reproductive Endocrinology Department of the Centro Médico Nacional 20 de Noviembre in Mexico City between 2015 to 2020. All couples underwent basic infertility tests, including day-3 FSH, LH, oestradiol, progesterone, prolactin and transvaginal pelvis ultrasound, and semen analysis for the male partner.

The inclusion criteria were as follows: all female patients who underwent an ovarian stimulation cycle for IVF/ICSI either with a mild ovarian stimulation protocol or a conventional stimulation protocol. Patients with double ovarian stimulation, incomplete medical records, oncologic patients undergoing IVF for fertility preservation, patients undergoing natural cycle and patients stimulated with clomiphene citrate were excluded.

The Ethics Committee approved the study protocol (Institutional Review Board, reference number 403.2022; approval date September 15th 2022). Written informed consent was waived owing to the study’s retrospective nature, and patient data were used anonymously.

### Ovarian stimulation protocols

Patients started ovarian stimulation on menstrual cycle day 2 or 3. If mild ovarian stimulation was performed, patients were given 5 mg of oral letrozole (Femara, Novartis Pharma Stein, Switzerland) with or without 150 UI recombinant FH (rFSH) (Gonal-F, Merck-Serono, Switzerland) plus 150 UI recombinant LH (rLH) (Luveris, Merck-Serono, Italy). When conventional ovarian stimulation was performed, the rFSH and rLH dosage was determined according to age, BMI value and previous ovarian response. Additionally, serum concentrations of FSH, LH, oestradiol and progesterone were determined, and transvaginal sonography (TVS) was carried out on that day. From stimulation day 8, routine TVS was carried out every two days to monitor follicle growth. The duration of rFSH varied according to follicular response. Patients should have at least three leading follicles with a mean diameter beyond 18 mm for triggering. In this study we only used HCG trigger, we exclude the protocols with agonist trigger. A 250mcg recombinant human chorionic gonadotrophin dosage was used subcutaneously (rhCG) (Ovidrel, Merck-Serono, Italy). All follicle measurements were carried out by experienced doctors using either a Voluson S10 Expert (GE Health Care, Parramatta, Australia) or a Clear Vue 350 (Philips, USA) with an intracavitary probe.

### Oocyte retrieval and fertilization

Oocytes were retrieved transvaginally 34-36 hours after rhCG administration. Oocytes were graded for maturity based on the morphological characteristics as previously described (Cortés-Vazquez *et al*., 2021).

### Outcome variables and definitions

Serum Progesterone on rhCG day (ng/ml), BMI (kg/m^2^), age, size, weight, number of retrieved oocytes, mature oocytes and oocyte maturation rate were recorded and analyzed. The primary outcome was to determine the correlation between progesterone levels on HCG day and oocyte maturation rate or the number of mature oocytes. A secondary outcome was to outline the patients with the lowest number of mature oocytes/ oocyte maturation rate after an IVF/ICSI cycle.

### Statistical analysis

A non-probabilistic convenience sampling was performed. A Spearman Rho analyzed variables that failed to have a normal distribution, otherwise were analyzed by a Pearson’s coefficient. Continuous data were verified for normalcy using the Kolmogorov-Smirnov test.

After that, simple linear regression and principal component analyses were performed. A Principal Component Analysis was performed to outline the group of patients with the highest and lowest oocyte maturation rates. All patients were divided into groups according to their progesterone level on HCG day (Group A <1.5ng/ml and Group B > 1.5ng/ml). A two-sided *p*-value of <0.05 was accepted as statistically significant. Data are presented by the mean with the corresponding standard deviation. Statistical analysis was conducted using SPSS version 23 (IBM Corp., USA).

## RESULTS

A total of 646 patients completed the inclusion criteria, and after discarding patients with incomplete records, 600 patients were eligible.

At Table 1, we show our patient’s demographic and clinical information. The mean age of our patients is 35 years (18 to 35 years), with a mean BMI of 26 kg/m^2^ (18 to 36), 6.4 oocytes retrieved, 4.32 mature oocytes per oocyte retrieval, a 63% maturation rate and mean 1.06ng/ml progesterone level on hCG day. The Kolmogorov-Smirnov test failed to demonstrate normalcy in our data. The Spearman Rho showed that estradiol and the number of mature oocytes positively correlate with progesterone levels on HCG day (Table 2). Progesterone levels on hCG day had a positive weak, and non-statistically significant correlation with oocyte maturation rates. Age revealed a weak negative correlation with progesterone levels on HCG day.

**Table 1:** Patient’s demographic and cycle parameters information.

Variables	Mean
Age (y)	35.7±3.52
Weight (kg)	65.6±10.65
Height (m)	1.58±0.06
BMI (kg/m^2^)	26.0±3.64
# Oocytes Retrieved	6.43±4.75
# Mature Oocytes	4.3±3.52
Oocyte Maturation Rate (%)	63.16±29.24
Progesterone on HCG day (ng/ml)	1.06±1.10
Oestradiol on HCG day (pg/ml)	2682.78±5701.20

Data are presented as mean ±SD. BMI, body mass index, HCG, human chorionic gonadotrophin.

**Table 2:** Correlations between ovarian response and progesterone level on HCG day.

Variables	Spearman coefficient (r)	p-value
Age	-0.083	0.043
BMI	-0.177	0.000
Oestradiol on HCG day	0.387	0.000
# Oocytes Retrieved	0.270	0.000
# Mature Oocytes	0.251	0.000
Oocyte Maturation Rate	0.025	NS

**Figure 1:**
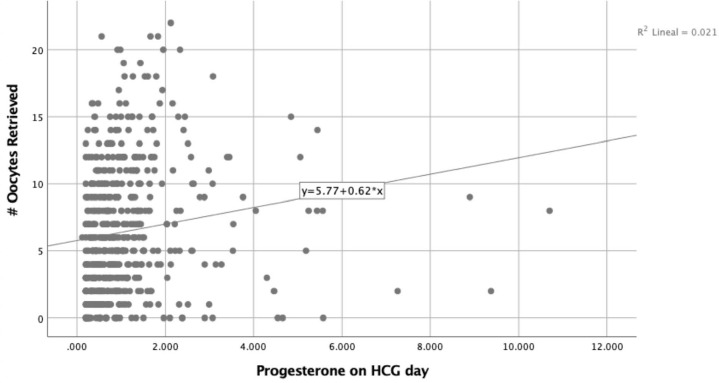
Progesterone levels on HCG day and # Oocytes Retrieved.

**Figure 2:**
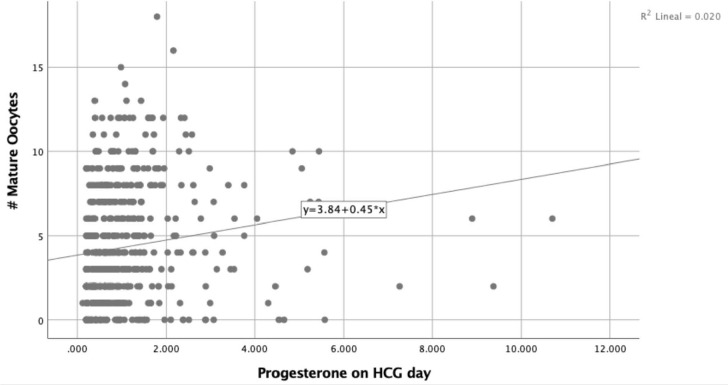
Progesterone levels on HCG day and # Mature Oocytes.

**Figure 3:**
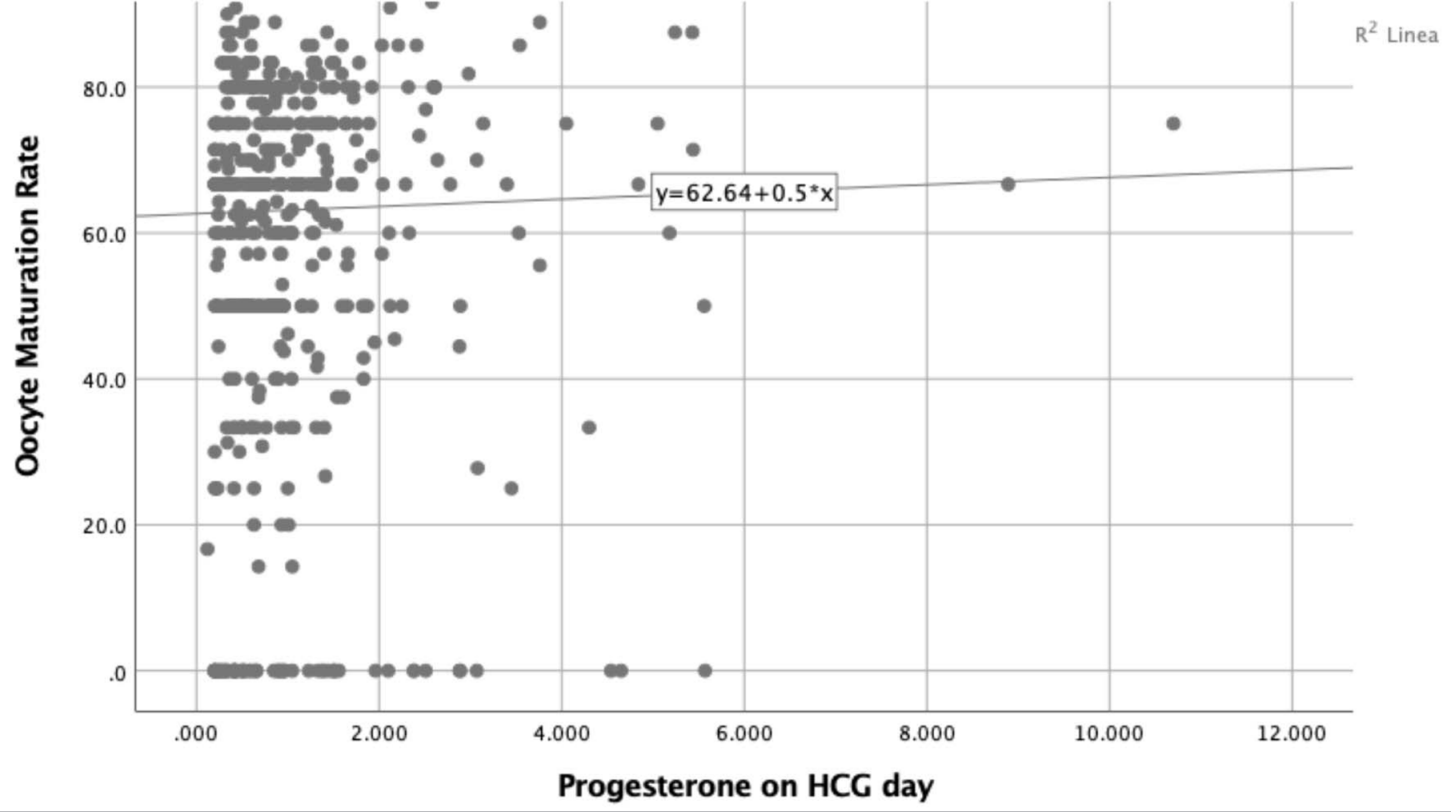
Progesterone levels on HCG day and # Oocytes maturation rate.

A simple linear regression model showed that higher progesterone values are associated with an increasing number of oocytes retrieved and mature oocytes as can be seen in Figures 1 and 2. Nevertheless, the simple linear regression model showed a powerless and non-statistically significant effect of progesterone levels on HCG day and oocyte maturation rates (Figure 3).

**Figure 4:**
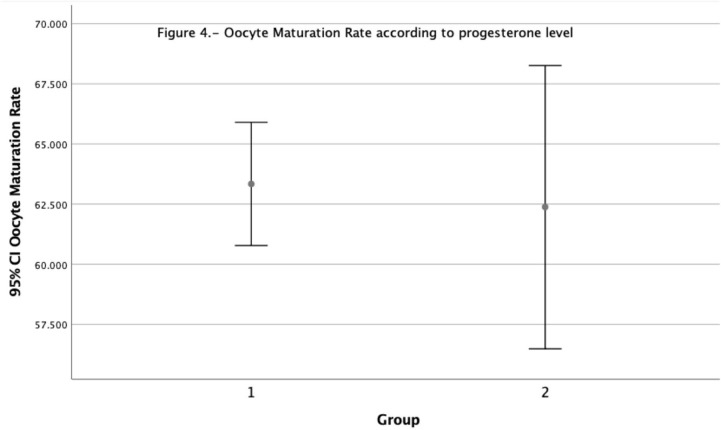
Oocytes Maturation Rate according to progesterone level.

A PCA with a Varimax rotation was performed (Table 3). The PCA displayed that using four components, we can explain an eighty-eight eigenvalue. The young patients distinguish component one, showing a normal ovarian response, high estradiol levels on HCG day, a high number of mature oocytes and low progesterone levels on HCG day. Component 4 is distinguished by low ovarian responders, low estradiol levels on HCG day, few mature oocytes and higher progesterone levels on HCG day. While patients in component 3 were older, with the lowest ovarian response, lowest mature oocytes and lowest progesterone levels on HCG day. It is essential to consider that component 2 had the highest maturation rates. After classifying patients according to their progesterone levels, we applied a Student’s T-test. All patients were classified according to their progesterone levels on HCG day, Group 1 patients with progesterone levels <1.5ng/ml and Group 2 >1.5mg/ml. There was no statistically significant difference between the two groups regarding oocyte maturation rate, as can be observed on Figure 4 and Table 4. Although Group 2 had a higher number of oocytes retrieved and a higher estradiol level.

**Table 3:** Principal component analysis.

	Component 1	Component 2	Component 3	Component 4
Age	-0.086	-0.015	0.963	-0.020
# Oocytes Retrieved	0.859	0.203	-0.267	0.038
# Mature Oocytes	0.814	0.457	-0.214	0.043
Progesterone levels on HCG day	0.096	0.013	-0.021	0.994
Oocyte Maturation Rate	0.097	0.954	-0.004	0.010
Oestradiol levels on HCG day	0.780	-0.168	0.180	0.107

**Table 4:** Comparison of oocyte maturation rates in patients with and without premature progesterone rise.

	Group 1 < 1.5 ng/ml (n=495)	Group > 1.5 ng/ml (n=105)	*p*-value
Age (y)	35.7±3.5	35.6±3.3	0.709
Weight (kg)	65.9±10.8	64±9.7	0.082
Height (m)	1.58±.06	1.58±0.07	0.614
BMI (kg/m^2^)	26.1±3.6	25.5±3.5	0.103
# Oocytes retrieved	6.03±4.3	8.3±5.9	0.000
# Mature oocytes	4.04±3.1	5.6±4.5	0.000
Oocyte maturation rate (%)	63.4±28.9	61.6±30.8	0.551
Progesterone levels on HCG day (ng/ml)	0.695±.353	2.799±1.668	0.000
Oestradiol levels on HCG day (pg/ml)	2160.55±4165.9	5144.73±9866.9	0.003

Data are presented as mean +/-SD. BMI, body mass index, HCG, human chorionic gonadotrophin.

## DISCUSSION

The primary objective of our study is to determine whether the serum progesterone levels on HCG day influence the number of mature and oocyte maturation rates. We discovered that the number of retrieved and mature oocytes positively correlates with progesterone levels on HCG day. Our secondary outcomes measures are similar to those reported by other authors, which show that progesterone levels beyond 1.5ng/ml are associated with higher serum oestradiol levels and a higher number of follicles (Kyrou *et al*., 2012).

Nevertheless, in the Spearman Rho test and simple linear regression model, progesterone levels on HCG failed to impact oocyte maturation rates significantly. Even age does not critically influence oocyte maturation rates based on our findings. These findings partially agree with a prior study at our institution (Cortés-Vazquez *et al*., 2021). Additionally, our previous experience could only reflect the patients’ outline corresponding to low ovarian responders.

Furthermore, our PCA test showed that high responders (component 1 of PCA) tend to have higher oestradiol and progesterone levels than patients in components 2 or 3. We speculate that continuously elevated FSH levels can explain this higher hormonal profile, so the number of precursor steroids generated may exceed the ability of the ovary to convert them into estrogen pathways (Lawrenz *et al*., 2018). So it is reasonable to believe that higher ovarian response will lead to higher progesterone levels and more mature oocytes compared to low responders.

As previously mentioned by other authors, the P4/E2 is a well-known marker of premature luteinization (Wu *et al*., 2015). Wu *et al*. (2015) demonstrated an age-related functional decline in granulosa cell function consistent with premature luteinization. This imbalance suggests accelerated luteinization, particularly in low ovarian responders (Younis, 2019). Indeed, older patients might be at higher risk for premature luteinization than younger patients (Wu *et al*., 2015). Our findings are consistent with this evidence since patients in components 3 and 4 exhibits lower ovarian responses and low oestradiol levels. They tend to have higher progesterone levels than other groups, demonstrating the functional decline in granulosa cell function. Also, our patients in components 3 and 4 will exhibit fewer retrieved and mature oocytes. Other groups reached similar conclusions regarding the hormonal profile of low ovarian reserve (Luborsky *et al*., 2002; Younis *et al*., 2001).

Meanwhile, component 2 patients have the highest oocyte maturation rate, lower than component 3/4 or 1 progesterone levels and low oestradiol production. We assume this group of patients could be those normal ovarian responders who underwent mild ovarian stimulation protocols. Since these patients have low oestradiol levels, it is feasible to believe that letrozole-induced ovarian stimulation could yield a more balanced number of mature/immature oocyte cohorts during oocyte retrievals.

The variety of ovarian stimulation protocols and ovarian responses to assisted conception techniques influences the number and oocyte maturation rates more than serum progesterone levels on HCG day. Even though higher progesterone values (>1.5ng/ml) were associated with an increased number of mature oocytes (as shown on Student’s T-test), this outcome does not reflect the reality of patients with low ovarian response. It will only reflect the reality of high-responder patients.

The clinical impact of these results is that clinicians must implement new strategies in their practice to increase oocyte maturation rates in low ovarian responders. Rescue in vitro maturation could be an option in proper centres to reduce cancellation rates due to the absence of transferable embryos. However, implantation rates after in-vitro rescue maturation remain low (Braga *et al*., 2010). Additionally, available evidence shows other strategies to improve the maturation rates in low responders. In a fascinating study, Zhang *et al*. (2017) showed a statistically significant higher maturation rate in low responders with a dual trigger, composed of a gonadotrophin-releasing hormone agonist and a standard dose human chorionic gonadotrophin. Another strategy that clinicians can offer to low responders is double ovarian stimulation. Evidence points out that luteal phase stimulation increases the number of oocytes available for fertilization while reducing the time required to obtain a euploid embryo and with acceptable ongoing pregnancy rates (Alsbjerg *et al*., 2019; Madani *et al*., 2019; Cerrillo *et al*., 2023). There is controversy on the optimal follicular size and oocyte maturation rates; what is clear is that larger follicular size is associated with higher maturation rates; in a prospective study, Mehri *et al*. (2014) showed a 99% maturation rate with follicular diameters beyond 18mm. So in the author’s opinion, it is a reasonable option to perform the hCG trigger in patients with leading follicles beyond 18 mm, above all in low ovarian responders (Mehri *et al*., 2014).

## CONCLUSIONS

Higher serum progesterone levels are associated with increased retrieved and mature oocytes in high responders. At the same time, higher progesterone levels in lower responders are not associated with optimal ovarian response.
